# An analysis of depressive symptoms in stroke survivors: verification of a moderating effect of demographic characteristics

**DOI:** 10.1186/s12888-017-1292-4

**Published:** 2017-04-08

**Authors:** Eun-Young Park, Jung-Hee Kim

**Affiliations:** 1 Department of Secondary Special Education, College of Education, Jeonju University, PO Box 560-759, 45 Baengma-gil, Wansan-gu, Jeonju, Korea; 2 Department of Clinical Nursing, College of Nursing, The Catholic University of Korea, 222 Banpo-daero, Seocho-gu, Seoul, 06591 Korea

**Keywords:** Depression, Demographic characteristic, Moderating effect, Stroke

## Abstract

**Background:**

The rehabilitation of depressed stroke patients is more difficult because poststroke depression is associated with disruption of daily activities, functioning, and quality of life. However, research on depression in stroke patients is limited. The aim of our study was to evaluate the interaction of demographic characteristics including gender, age, education level, the presence of a spouse, and income status on depressive symptoms in stroke patients and to identify groups that may need more attention with respect to depressive symptoms.

**Methods:**

We completed a secondary data analysis using data from a completed cross-sectional study of people with stroke. Depression was measured using the Center for Epidemiologic Studies Depression Scale.

**Results:**

In this study, depressive symptoms in women living with a spouse were less severe than among those without a spouse. For those with insufficient income, depressive symptom scores were higher in the above high school group than in the below high school group, but were lower in patients who were living with a spouse than in those living without a spouse.

**Conclusion:**

Assessing depressive symptoms after stroke should consider the interaction of gender, economic status, education level, and the presence/absence of a spouse. These results would help in comprehensive understanding of the importance of screening for and treating depressive symptoms during rehabilitation after stroke.

## Background

Depression is reportedly the most common mental disorder following stroke [1–4], with an incidence ranging from 10 to 64% [4–6]. Poststroke depression has an adverse effect on functional recovery and increases the mortality rate [7]. Furthermore, the rehabilitation of depressed stroke patients is more difficult because poststroke depression is associated with disruption of daily activities, function, and quality of life [8, 9].

Depressive symptoms may be a psychological consequence of low levels of physical functioning and different life experiences after a stroke [10]. Indeed, one study found that there was no significant association between depression levels and the pathological development of stroke such as type of stroke, side of stroke, and comorbidity [10]. Furthermore, a study on comorbidity and primary care costs found that depression was a stroke comorbidity associated with increased costs [11].

Demographic characteristics are one of factors that affect depressive symptoms in stroke patients. A meta-analysis in stroke patients reported that education level, income, and age showed significant effects on depressive symptoms [12]. Other studies have found that depressive symptoms are significantly related to presence of a spouse [13] and higher levels of education [14], while low economic status has also been associated with a higher prevalence of depression at any level of morbidity [15]. Stroke patients were also shown to receive care and emotional and physical support from their family members [16]. Support from the family was a protective factor affecting poststroke depression [17]. Indeed, the severity of depressive symptoms has been found to be associated with lower levels of certain relational variables, such as social support and satisfaction thereof [18]. The literature has shown that depressive symptoms can be reduced through interventions that aim to improve the self-concept and self-esteem, such as positive thinking, as well as social/family support [19–21]. Functional independence was also found to be a crucial factor contributing to the level of depressive symptoms [22]. Overall, the literature has found evidence for relationships between gender, age, education level, presence of a spouse, income status, and poststroke depressive symptoms.

Demographic characteristics including gender roles, family role, caregiving support, and socioeconomic status make up a shared environment and are interdependent and interaction dynamics [23–25]. There is, however, some controversy regarding the relationships between demographic characteristics and depressive symptoms: namely, one study found no significant differences or correlations between depression score and these demographic characteristics [17]. Given the chronic nature and long-term care after stroke, it seems necessary to precisely understand how depressive symptoms result from this pathology, which can be done through attending to the moderating effects of demographic variables on psychological status.

To our knowledge, little is known about the potential role of demographic factors in depression following a stroke. The aim of our study was to evaluate the interaction of demographic characteristics including gender, age, education level, the presence of a spouse, and income status on depressive symptoms after stroke and to identify groups of patients that may be at risk of depressive symptoms after stroke.

## Methods

### Participants

We completed a secondary data analysis using data from a completed cross-sectional study in stroke patients. The sample was a convenience sample derived from a study that aimed to create a model for participation restriction in chronic stroke survivors [26]. Written informed consent was obtained from all participants and the study was reviewed and approved by the Ethic Review Board of Y University in Korea. Inclusion criteria for this study were (1) having a confirmed diagnosis of stroke based on hospital records, (2) experiencing their first stroke based on hospital records, (3) having a stroke diagnosis at least 12 months prior to data collection, (4) having sufficient communication and cognitive abilities to answer questions, and (5) living in the community. Cognitive function was assessed by using the validated Korean version of the Mini-Mental State Examination (MMSE-K) [27]; the scores can range from 0 to 30, with higher scores indicating better cognition. In this study, subjects whose MMSE-K scores were 18 and below were excluded.

### Measures

#### Depressive symptoms

Depressive symptoms were measured using the Center for Epidemiologic Studies Depression Scale (CES-D). The reliability and validity has been verified in stroke patients [28, 29] and this scale is often used in studies of stroke outcomes [30, 31]. The CES-D scale consists of 20 items scored on a four-point Likert scale ranging from 0 (rarely or none of the time) to 3 (most or all the time); four items are reverse scored. The total score is calculated by summing the item scores. The Korean version of the CES-D scale has total scores ranging from 0 to 60 points. Higher scores indicate more severe depressive symptoms, with a score of 16 or greater suggesting the presence of clinical depressive symptoms [32]. The Cronbach’s α coefficient in stroke patients has been found to be 0.87 with a 95% confidence interval of 0.84–0.89 [29].

#### The presence of a spouse

Marital status was classified as the presence or absence of a spouse (including not married, divorced, widowed, and separated). The answers were then recoded into a dichotomous variable whether the spouse lived with the stroke patient or not.

#### Income status

Income status was classified as sufficient and insufficient income categories. This variable was measured subjectively by asking participants, “What was your economic status?” Participants answered on a five-point scale with answer options of “very sufficient,” “sufficient,” “average,” “somewhat insufficient,” and “very insufficient.” Participants’ answers were then recoded into a dichotomous variable indicating whether participants had experienced subjective income hardship or not. Average or above income was classified as “sufficient income” and “somewhat insufficient” and “very insufficient income” were classified as insufficient income.

### Statistical analysis

The mean differences in the demographic characteristics were analyzed using independent t tests. The general linear model (GLM) is a generalization of multiple linear regression models that can flexibly measure the relationship between normally distributed dependent variables and some combination of categorical or continuous independent variables. The GLM can be used to analyze the mean differences when there is more than one independent variable. The results of the GLM can reveal the moderating effects among the independent variables. In this study, we used the univariate GLM (which involves conducting an analysis of variance for variables with two or more factors) because there was one dependent variable. A full factorial design was selected and type III sums of squares were used [33]. The parametric tests were used from an ordinal scale of the CES-D in data because Likert scales are often viewed as an interval scale [34].

Here, we determined whether there were significant main effects of gender, education level, age, income status, and the presence of a spouse on depressive symptoms. We also examined the interactions between gender, education level, age, income status, and the presence of a spouse. Specifically, we measured the interactions of gender × education level, gender × age, gender × income status, education level × age, education level × the presence of a spouse, education level × the presence of a spouse, and education level × income status, age × the presence of a spouse, age × income status, and the presence of a spouse × income status on depressive symptoms. The statistical analysis was performed using SPSS Statistics 23.0.

## Results

### Characteristics of the participants

Data from 166 stroke patients (57 women and 109 men) were analyzed. Their mean age was 53.40 years (SD = 13.47). The general characteristics of the patients and the group differences are presented in Table 1. There were no significant differences in the number of participants according to age or presence of spouse. There were, however, significantly more male participants, participants with an education level of above high school, and participants with a sufficient income.

**Table 1:** General characteristics of participants

Category	n	M	SD	t	p
Gender					
Male	109	19.38	10.86	−2.12	**.036**
Female	57	23.28	12.05		
Age					
< 60	120	20.92	10.85	.36	.717
≥ 60	46	20.20	12.84		
the presence of spouse					
yes	108	20.22	11.35	−.76	.447
no	58	21.64	11.54		
Education					
Below high school	47	21.26	12.64	2.56	**.012**
Above high school	119	19.32	10.61		
Household income					
Sufficient income	120	18.43	11.40	−4.39	**.000**
Insufficient income	46	26.67	9.11		

### Moderating effects of demographic characteristics on depression

Patients’ mean scores on the CES-D according to their demographic characteristics are presented in Table 2. The depressive symptoms score was not associated with education or the presence of a spouse. However, females, participants with less than a high school education, and participants with an insufficient income had significantly higher depressive symptom scores. The main effects of gender, age, education level, presence of a spouse, and income status on depressive symptoms are presented in Table 2. The main effects of gender and age were significant.

**Table 2:** The moderating effects of general characteristics on depression

	df	SS	F	p
Gender (male/female)	1	666.794	6.645	**.011**
Education level (below high/above high)	1	.107	.001	.974
Age (< 60/≥ 60)	1	417.839	4.164	**.043**
Income status (general/low)	1	34.242	.341	.560
The presence of spouse (yes/no)	1	381.870	3.805	.053
Gender × Education level	1	25.642	.256	.614
Gender × Age	1	2.099	.021	.885
Gender × The presence of spouse	1	493.127	4.914	**.028**
Gender × Income status	1	165.172	1.646	.202
Education level × Age	1	.749	.007	.931
Education level × The presence of spouse	1	22.879	.228	.634
Education level × Income status	1	496.013	4.943	**.028**
Age × The presence of spouse	1	78.361	.781	.378
Age × Income status	1	17.688	.176	.675
The presence of spouse × Income status	1	413.696	4.123	**.044**

**Fig. 1:**
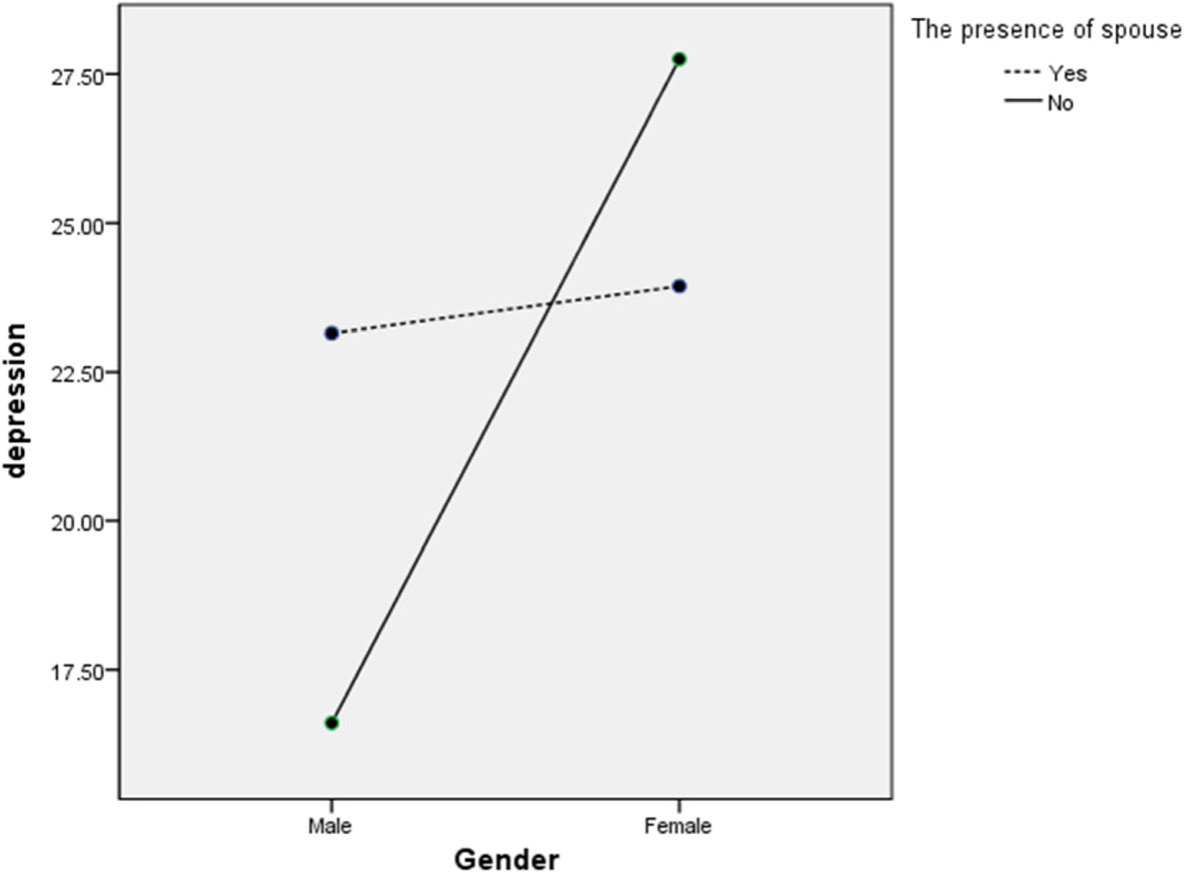
Interaction effect of gender × the presence of spouse on depression

**Fig. 2:**
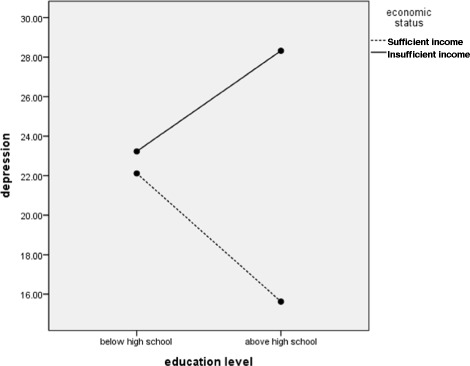
Interaction effect of education level × income status on depression

**Fig. 3:**
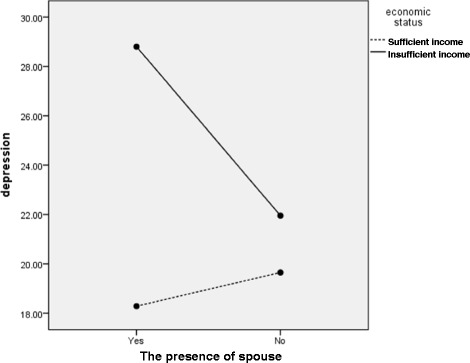
Interaction effect of the presence of spouse education level × income status on depression

The interaction effects of gender × presence of a spouse, education level × income status, and presence of a spouse × income status on depressive symptoms were also significant. More specifically, the depressive symptom scores of women living with a spouse were significantly lower than were those of women without a spouse (Fig. 1). Furthermore, among participants with an insufficient income, the depressive symptoms scores were higher among those in the above high school group than among those in the below high school group. Furthermore, among participants in the above high school group, depressive symptom scores were lower (16.48) in the sufficient income group than in the insufficient income group (27.73). There was a trivial difference in depressive symptoms by income status among participants in the below high school group (insufficient income, 24.03; sufficient income, 24.69; Fig. 2). Finally, in the insufficient income group, the depressive symptoms of stroke patients living with a spouse were more severe than were those of patients living without a spouse. Conversely, economic status had a larger effect on stroke patients living with a spouse (Fig. 3).

## Discussion and conclusion

In this study, we investigated the relationships of various demographic characteristics—including gender, age, education level, income status, and the presence of a spouse—with depressive symptoms among stroke patients, as well as the interaction effects of these variables.

In this study, the depressive symptoms scores of women living with a spouse were lower than were those of women without a spouse. By contrast, the difference in depressive symptoms scores according to the presence of a spouse was trivial among male patients. Past studies have similarly shown gender-based differences in stroke outcomes, including daily activity independence and quality of life [35]. Female stroke patients appear to be less likely to achieve independence in daily activities and have poorer physical, cognitive, and emotional functioning (manifesting as problems such as thinking difficulties, language difficulties, and less energy) after discharge [36]. Considering that spouses are the primary caregivers for most patients are physically dependent on others [37], it is possible that there are partner effects on emotional functioning, such as the development of depressive symptoms, among female stroke patients.

Family support has been reported as a protective factor for major depression in chronic diseases such as cancer [38]. Research has similarly shown that depressive symptoms are negatively correlated with social support [17]; thus, it is logical to suggest that stroke patients who do not have a spouse might be more depressed than those with a spouse. In terms of the social contextual model, which accounts for the transactional and interdependent nature of social relationships, a diagnosis of chronic illness might lead to changes that influence one’s partner as well [23–25]. However, this model cannot explain why depressive symptoms were found among male stroke patients who had a spouse in our study. There are studies suggesting gender differences in partner effects [13]. Thus, our results are also supported by a study reporting that there are no partner effects in male patients. In other words, husbands’ depressive symptoms do not appear to be influenced by the wives’ characteristics, whereas the husband’s characteristics can contribute to women’s somatic symptoms and depressive symptoms [13].

In our results, the depressive symptoms of stroke patients with an education level above high school and insufficient income were higher than were those in the sufficient income group. Education has been found to be negatively associated with general depressive symptoms [13, 14, 39, 40], likely because of its relationship with future income, socioeconomic status, and life satisfaction [41]. However, education did not have a protective role in the insufficient income group; it was only protective in the sufficient group in our study. This might be because material deprivation is an important variable in stroke patients. Patients with stroke are long-term users of health services and typically have high health care costs [42]. Low income has been found to be associated with less participation following stroke [43]. Additionally, around 20% of stroke survivors are unemployed post-stroke and around half must change jobs [37]. Thus, under conditions of material deprivation, higher levels of education do not guarantee higher income. Because there was a strong association between low income and depression among patients [15], further studies should be conducted to examine economic status changes after a stroke.

Note that this study employed a subjective measure of income status. Subjective income status is an integral aspect of one’s economic well-being because it can improve one’s assessment of one’s capacity to meet financial needs, including maintaining independent community-based living [44]. Perceived income adequacy has been reported in the literature in various ways and has been verified as a predictor of other outcome measures such as self-rated health [45], life satisfaction [46], and depressive symptoms [47].

In the same line of reasoning, with insufficient income, the presence and support of a spouse did not have positive effects on depressive symptoms in stroke patients. In our study, the depressive symptoms scores of stroke patients living with a spouse in the insufficient income bracket were higher than were those living without a spouse. Overall decline in social function and burden for both the stroke survivors and their caregivers [37] would be much greater among those with a low income status.

Stroke is a complex condition that can have multiple comorbidities [48]. Because managing complex patients requires greater clinical effort, a better understanding of this complex patient population is needed [49]. The complexity of these patients increases the need for healthcare resources and substantial family and community support; in particular, healthcare systems and services need to be redesigned to better meet the needs of these patients [49]. Personal characteristics, social determinant factors, and social/family support have been reported as some of the elements that contribute to the complexity of stroke patients [48]. Because depressive mood leads to a lowered likelihood of help-seeking intention [50], identifying the risk factors of depressive symptoms after a stroke is necessary. This study showed that assessment of risk factors should consider the interactions between gender, economic status, education level, and presence/absence of a spouse. This would also be relevant for interventions aiming to reduce depressive symptoms during stroke rehabilitation.

The results of this study help in comprehensive understanding of the importance of screening for and treating depressive symptoms during rehabilitation after stroke. In particular, our results showed that the researcher took into account the interaction between general characteristics such as gender, socioeconomic status, and the presence/absence of a spouse. It might be noted, however, that researchers must be cautious in generalizing our findings, as the sample may not be fully representative of all stroke patients, especially those who are institutionalized or have cognitive deficits. Furthermore, our sample size was rather small, which increases the likelihood of spurious associations among a large number of interactions. Investigating such a large number of potential interactions indeed presents a statistical challenge for studies with relatively small sample sizes. Moreover, the probability of making a type II error could increase due to lack of adjustment for multiple comparisons. There was a need to be cautious when interpreting these results from the point of view that chance could increase with each subsequent test. Finally, the data were all collected through a self-report questionnaire, and some of the variables, in particular income status, were subjectively measured. These facts suggest that the validity of our results might have been influenced by social desirability bias and intrinsic self-reporting bias.

## Abbreviations

CES-D: Center for epidemiologic studies depression scale
EP: Eun-young Park
GLM: General linear model
JK: Jung-Hee Kim
MMSE-K: Mini-mental state examination
